# *Bacillus halotolerans* KKD1 induces physiological, metabolic and molecular reprogramming in wheat under saline condition

**DOI:** 10.3389/fpls.2022.978066

**Published:** 2022-08-11

**Authors:** Xiaohui Wu, Yaning Fan, Ruoyi Wang, Qian Zhao, Qurban Ali, Huijun Wu, Qin Gu, Rainer Borriss, Yongli Xie, Xuewen Gao

**Affiliations:** ^1^Key Laboratory of Integrated Management of Crop Diseases and Pests, Ministry of Education, Department of Plant Pathology, College of Plant Protection, Nanjing Agricultural University, Nanjing, China; ^2^State Key Laboratory of Plateau Ecology and Agriculture, Department of Grassland Science, College of Agricultural and Husbandry, Qinghai University, Xining, China; ^3^Institut für Biologie, Humboldt Universität, Berlin, Germany; ^4^Nord Reet UG, Greifswald, Germany

**Keywords:** *Bacillus halotolerans*, PGPR, genome analysis, salt stress, plant-stress response, plant physiological and metabolism

## Abstract

Salt stress decreases plant growth and is a major threat to crop yields worldwide. The present study aimed to alleviate salt stress in plants by inoculation with halophilic plant growth-promoting rhizobacteria (PGPR) isolated from an extreme environment in the Qinghai–Tibetan Plateau. Wheat plants inoculated with *Bacillus halotolerans* KKD1 showed increased seedling morphological parameters and physiological indexes. The expression of wheat genes directly involved in plant growth was upregulated in the presence of KKD1, as shown by real-time quantitative PCR (RT-qPCR) analysis. The metabolism of phytohormones, such as 6-benzylaminopurine and gibberellic acid were also enhanced. Mining of the KKD1 genome corroborated its potential plant growth promotion (PGP) and biocontrol properties. Moreover, KKD1 was able to support plant growth under salt stress by inducing a stress response in wheat by modulating phytohormone levels, regulating lipid peroxidation, accumulating betaine, and excluding Na^+^. In addition, KKD1 positively affected the soil nitrogen content, soil phosphorus content and soil pH. Our findings indicated that KKD1 is a promising candidate for encouraging wheat plant growth under saline conditions.

## Introduction

Plants are sensitive to different abiotic stress conditions, such as salinity, flooding, drought, cold, and heavy metals. Major yield losses may occur when crops are exposed to these stresses (Zhao et al., 2021). Due to climate change, the occurrence of salt stress, which negatively affects crop yield, is steadily increasing. It has been estimated that almost 20% of the total cultivated land worldwide is adversely affected by soil salinization (Yue et al., 2021). The predominant chemical compound in saline soil is NaCl (Chen et al., 2021). A high level of soil salinity inhibits the growth of numerous crop species, since most crops possess only poor salt tolerance (Julkowska and Testerink, 2015). Salt stress severely undermines crop growth and productivity by disturbing plant nutritional and hormonal processes (Yu et al., 2020). The extensive application of synthetic agrochemicals is considered to be responsible for the increase in soil salinity and the loss of soil nutrients (Galieni et al., 2020). Agrochemicals contain a large amount of Cl^–^, SO_4_^2–^, and other ions. These soluble salts do not merely deteriorate the fertility of the soil but also adversely affect plant development (Morales-Cedeño et al., 2021).

Wheat (*Triticum aestivum*) is a major cereal crop worldwide, with one-third of the human population depending on wheat as a main staple food (Shewry, 2009). Salinity causes ion toxicity, osmotic stress, and nutrient unavailability and also increases the generation of reactive oxygen species (ROS) and reduces the chlorophyll content (Manzoor et al., 2021). As a consequence of soil salinity, wheat plants have developed multiple defense mechanisms to combat salt stress. Based on these defense mechanisms, several molecular breeding and genetic engineering methods have been developed for improving wheat yield under salt stress. However, these methods are arduous and time consuming (Chenu et al., 2017). Animal manure is regarded as a safe and efficient organic fertilizer, but its frequent application causes soil salinization (Siddiqui and Futai, 2009). As a result of the above problems, a sustainable and eco-friendly approach that harnesses the beneficial characteristics of microorganisms from the plant rhizosphere appears to be a suitable way to enhance plant development (Arif et al., 2020).

Plant growth-promoting rhizobacteria (PGPR) are a diverse group of plant-associated microorganisms that are able to colonize plant roots, interact with plants, stimulate plant growth, and positively affect plant metabolism (Lugtenberg and Kamilova, 2009). Increased evidence has revealed that some PGPR possess strong stress tolerance and are able to alleviate plant stress damage through a wide range of strategies, such as strengthening plant antioxidant activities, reducing ion toxicity, and producing phytohormones (Ha-Tran et al., 2021). *Pseudomonas frederiksbergensis* strain OS261 can augment salt tolerance and promote red pepper plant growth through improving antioxidant activity and decreasing hydrogen ion concentration in the leaf tissues (Chatterjee et al., 2017). The psychrophilic *Bacillus* strains CJCL2 and RJGP41 improved plant growth under cold stress conditions (Zubair et al., 2019). *Bacillus flexus* KLBMP 4941 promoted the growth of the coastal halophyte *Limonium sinense* under salt stress by regulating Na^+^/K^+^ homeostasis (Xiong et al., 2020). *B. subtilis* GB03 confers salt tolerance in *Arabidopsis thaliana*. under salt stress, strain GB03 downregulates the HKT expression, thereby reducing the Na^+^ uptake and providing salt tolerance to plants (Zhang et al., 2008).

Among the beneficial bacteria, the gram-positive and spore-forming *Bacilli* can withstand harsh environments and also produce various metabolic products (Tzipilevich et al., 2021). Increasing evidence has revealed that plants are positively influenced by PGPR. However, the manner in which PGPR modulate plant gene expression is unclear, and the associated metabolic processes also need to be further clarified. In the present study, we evaluated the potential for the halophilic *Bacillus* strain KKD1 to alleviate salt stress in wheat plants.

The growth-promoting activity of *B. halotolerans* KKD1 was analyzed using pot experiments, and the plant metabolic changes initiated by KKD1 were assessed by high-performance liquid chromatography-mass spectrometry (HPLC-MS) and real-time quantitative PCR (RT-qPCR) analysis. Genome sequencing revealed that KKD1 encoded various genes related to PGP traits. KKD1 mitigated the adverse effects caused by salinity stress (NaCl 300 mM) on wheat plants. Furthermore, the soil physicochemical properties of the wheat rhizosphere under salt stress were markedly improved when treated with KKD1.

## Materials and methods

### Bacterial strain, wheat seeds, soil samples, and culture conditions

*Bacillus halotolerans* KKD1, stored in our laboratory, was used in this study. KKD1 was isolated from the rhizosphere of *Androsace umbellata* in Hoh Xil (34°19′N and 89°25′E), Qinghai, China. For routine growth, KKD1 was grown overnight in LB (Luria–Bertani) liquid medium (tryptone 1.0%, yeast extract 0.5%, and NaCl 1.0%, pH 7.0) with shaking at 200 rpm at 37°C. The variety of wheat used in the study was Yannong 0428. The rhizosphere soil sample (0.5 kg) was collected from the wheat root surfaces, air dried, passed through a 2-mm sieve to eliminate plant material, and used for the determination of physical characteristics using standard procedures.

### Indole-acetic acid production assay

*In vitro* indole-acetic acid (IAA) production of KKD1 was examined by the Salkowski colorimetric method (Gang et al., 2019). KKD1 was cultivated in LB liquid medium [containing 0.1% (*w/v*) tryptophan] for 7 days and then centrifuged (4°C, 10,000 rpm, 10 min) to separate the culture broth from the cells. One milliliter supernatant was mixed with the same amount of colorimetric solution (30 mL of 0.5 mol/L FeCl_3_ + 30 mL sterile water + 50 mL of 98% H_2_SO_4_), the light absorption value of the solution was measured at OD_530_, and the amount of IAA was calculated by comparison with the standard curve. Three independent experiments were conducted to avoid artifacts caused by handling (Zhang C. et al., 2022).

### Cellulose degradation activity

The cellulose degradation activity was assessed by the staining method. KKD1 was cultivated in LB liquid medium overnight, following which 5 μL of bacterial suspension (OD_600_ = 1.0) was inoculated onto CMC (Carboxymethylcellulose) sodium medium [6 g NaCl, 0.1 g MgSO_4_⋅7H_2_O, 0.5 g KH_2_PO_4_, 0.1 g CaCl_2_, 2 g K_2_HPO_4_, 15 g CMC-Na, and 2 g (NH_4_)_2_SO_4_] and incubated at 37°C. After 48 h, the CMC medium was stained by Gram iodine (Gohel et al., 2014), and the formation of clearance zones around colonies was considered as enzymatic activity. The size of the clearance zones indicated the cellulose degradation ability of KKD1. Three independent experiments were conducted to avoid artifacts caused by handling (Zang et al., 2015).

### Protease activity assay

The production of protease in KKD1 was determined using skim milk agar according to the Safford and Stark method (Hadjidj et al., 2018). Protease production was tested on agar medium containing (g/L): 20-g skim milk powder and 20-g agar. KKD1 was cultivated in LB liquid medium overnight, following which 5 μL bacterial suspension (OD_600_ = 1.0) was inoculated onto the medium and then incubated at 37°C for 48 h. The size of the clearance zones indicated the protease degradation ability. All experiments had three replicates.

### Amylase activity assay

To measure amylase activity, KKD1 was cultivated in LB liquid medium overnight, following which 5 μL bacterial suspension (OD_600_ = 1.0) was inoculated onto starch containing medium (5-g yeast extract, 10-g NaCl, 10-g starch, and 20-g agar) and incubated at 37°C. Potassium iodine staining was performed after 48 h. The size of the clearance zones indicated the amylase activity of KKD1. Three independent experiments were conducted (Pan, 2021).

### Bio-surfactant assay

Bio-surfactant activity was analyzed using the expelling oil method. Ten milliliters of the light fraction of paraffin oil (CAS: 8042-47-5, Sangon Biotech Co., Ltd., China) was stained red using 10 mg Sudan III dye (CAS: 85-86-9, Beijing Solarbio Science and Technology Co., Ltd.). One milliliter of the red oil together with 20 mL double-distilled water was added onto the petri plate. After the red oil had spread across the entire plate surface, 1 μL crude extract was dropped in the center of the petri plate, and 1 μL methanol was used as a control. Three independent experiments were conducted (Liang et al., 2020).

### Swarming motility assay

A swarming motility assay in semisolid agar plates (0.7% agar concentration) was conducted based on the procedure described previously (Fraser and Hughes, 1999). Strain KKD1 was first grown in tubes with basal LB liquid medium and then incubated at 37°C and 180 rpm until OD_600_ = 1.0. Aliquots of 5 μL of each culture were seeded at the center of the swarming plates and then incubated at 37°C. The migration halo was monitored over 12–24 h, and the swarming diameters were measured. Three independent experiments were conducted.

### Determination of biofilm formation

The formation of biofilm by KKD1 was determined using Msgg (minimal salts glycerol glautamate) medium (5 mM potassium phosphate, 100 mM Mops, 2 mM MgCl_2_, 700 μM CaCl_2_, 50 μM MnCl_2_, 50 μM FeCl_3_, 1 μM ZnCl_2_, 2 μM thiamine, 0.5% glycerol, 0.5% glutamate, 50 μg/mL tryptophan, 50 μg/mL phenylalanine, and distilled water to 1,000 mL) in 12-well polystyrene plates (Corning Display Technology (China) (Kampf et al., 2018). The strain KKD1 was first grown in tubes with LB liquid medium, incubated at 37°C, 180 rpm until OD_600_ = 1.0, following which 150 μL of bacterial suspension mixed with 4 mL of MSgg medium, and the resultant were transferred to 12-well polystyrene plates and then tightly sealed and further incubated at 37°C. Biofilm formation was observed in 12 well plates after 2–4 days later (Fan et al., 2018).

### Genomic scanning of plant growth promotion traits in KKD1

Whole genome information of *B. halotolerans* KKD1 was analyzed on the Majorbio Cloud Platform^1^ and GO (Gene Ontology) database.^2^

### Plant growth promotion traits of KKD1 on wheat plants under greenhouse and saline conditions

Pot experiments was used to evaluate the growth-promoting effect of *B. halotolerans* KKD1. KKD1 was cultured in LB medium and incubated with shaking (200 rpm) for 12 h at 37°C. Then the cells were recovered by centrifugation and resuspended in the sterilized water until a final OD_600_ of 1.0 (1 × 10^7^ cfu/mL). The wheat seeds were randomly selected, randomly divided into different groups, surface sterilized with 15% sodium hypochlorite for 10 min and 75% alcohol for 30 s, and then finally rinsed with sterilized water. Thereafter, the seeds were soaked in the bacterial suspension for 4 h. The seeds were sown in basins and cultured in the greenhouse (25°C, photoperiod 16 h/8 h) for 15 days until subsequent experimental analysis. Sterile water treatments were used as the control, and 30 seeds were used for each treatment (Xie et al., 2014).

A pot experiment was conducted to evaluate the PGP traits of *B. halotolerans* KKD1 on wheat plants under salt stress. The method is the same as the treatment in the greenhouse, The seeds were soaked in the bacterial suspension for 4 h, sown in basins, and cultured in a greenhouse (25°C, photoperiod 16 h/8 h) for 5 days. From the 6th day, the wheat seeds were irrigated with sterilized water and one of three salt solutions (100, 200, and 300 mM) every 5 days and then cultured in the greenhouse for 20 days for subsequent experiment analysis. Sterile water treatments were used as the control, and 30 seeds were used for each treatment (Liu et al., 2020).

### Determination of peroxidase and catalase activities in the wheat plants

The catalase (CAT) activity was measured by a CAT assay kit (BC0200, Solarbio, Beijing, China) and the peroxidase (POD) activity was measured by a POD assay kit (BC0090, Solarbio, Beijing, China) according to the manufacturer’s instructions (Boamah et al., 2021).

### Measurement of lignin, total sugar, and glycine betaine contents in the wheat plants

The content of lignin was measured using a Lignin Content Test Kit (MZS-2-G, Suzhou Comin Biotechnology Co., Ltd., China) according to the manual. The total sugar was measured using the sulfuric acid-phenol method, as described previously. The glycine betaine content was measured using a glycine betaine assay kit (BC3130, Solarbio, Beijing, China) according to the manufacturer’s instructions (Tao et al., 2019).

### Determination of the Na^+^ concentration in wheat plants

The sodium content in the wheat seedlings was determined following Fu et al. (2022). The concentration of Na^+^ in the wheat seedlings was measured after diluting with 2% HNO_3_ (v/v) by inductively coupled plasma-mass spectrometry (ICP-MS, Agilent 7900, Agilent Technologies, Santa Clara, CA, United States).

### Quantification of endogenous phytohormone production by high-performance liquid chromatography-mass spectrometry analysis

The endogenous phytohormones [6-benzylaminopurine (6-BA), isopentenyl adenosine (iPA), Gibberellic acid 3 (GA3), Gibberellic acid 7 (GA7), and salicylic acid (SA) in the wheat plants were detected by HPLC-MS. Extraction was performed in the following steps: (1). Each wheat seedling sample (1.0 g) was ground into a powder with a pestle and mortar in liquid nitrogen; (2). The ground samples were homogenized in 10 mL acetonitrile and kept at 4°C; (3). The samples were centrifuged at 10,000 *g* for 5 min at 4°C, followed by the collection of supernatants of each sample in new tubes; (4). The supernatants were dissolved with five times the volume of acetonitrile solution and extracted twice, and the supernatants were merged; (5). The supernatant was filled with 35 mg C18 packing (CNWBOND HC-C18, ANPEL Laboratory Technologies, Shanghai, Inc.), and the samples were shaken for 30 s and then centrifuged at 10,000 *g* for 5 min, following which the supernatants were collected; (6). The supernatants were dried through vacuum evaporation, and each sample was dissolved in 200 μL methanol, filtrated through a 0.22-μm organic phase filter, and subjected to HPLC (Agilent 1290 Infinity, Agilent Technologies, Santa Clara, CA, United States) and a quantitative screening mass spectrometry system (AB SCIEX QTRAP 6500 System, AB Sciex, United States) for endogenous hormone detection and quantification (Allwood and Goodacre, 2010).

### Standard curves of the different metabolites

In total, 80 μL of each phytohormone mother liquor (10,000 ng/mL) was added into 2-mL centrifuge tubes and then homogenized in 2 mL of chromatographic grade methanol (Merck KGaA, Darmstadt, Germany), and standard solutions with a final concentration of 200 ng/mL were obtained. The standard solution (200 ng/mL) was then diluted with chromatographic-grade methanol (Merck KGaA, Darmstadt, Germany) to nine different concentrations, including 0.1, 0.2, 0.5, 1, 2, 5, 10, 50, and 200 ng/mL. Finally, the standard curves were based on these nine concentrations of standard liquid as shown in figures in Supplementary materials 1, 2.

### Expression profiling of growth-related genes in wheat plants by real-time quantitative PCR

The total RNA of the wheat leaves was extracted from different samples using a Plant RNA Rapid Extraction Kit (Coolaber Technology Co., Ltd., Beijing, China). The concentration and purity of the RNA were verified by measuring the absorbance at 260/280 nm. The first-strand cDNA was synthesized using the OneScript^®^ cDNA Synthesis Kit (ABM, Applied Biological Materials, Inc., Zhenjiang, China). The synthesized cDNA was used as a template for RT-qPCR. The primers were designed by the Primer Quest tool of IDT^3^ (Supplementary material 3). The SYBR Green qPCR master mix (Takara Bio, Beijing, China) was used for PCR reactions, and the expression of genes was studied in a Real-time Thermocycler (QuantStudio-6 Thermo Fisher Scientific, San Jose, CA, United States) using a cycling program of 95°C for 30 s, 40 cycles of 95°C for 5 s, and 34 s at 60°C. The relative expression levels of all samples were calculated and analyzed based on the 2^–ΔΔCT^ method (Zubair et al., 2019).

### Soil analysis

Soil samples were air-dried at room temperature until obtaining a solid mass, following which they were crushed and sieved to separate the fractions < 2 mm from gravel or larger detritus. The following soil properties and components were determined: soil total nitrogen, soil available nitrogen, soil total phosphorus, and soil pH. The semi-micro Kjeldahl digestion procedure was used to measure the soil total nitrogen concentration (Fawcett, 1954). Soil available nitrogen was assayed according to the alkali-hydrolytic diffusion method. Soil total phosphorus concentration was determined by using the molybdenum blue method. The pH of each soil sample was measured at a soil: water ratio of 1:2.5 in distilled water using pH meter (Tao et al., 2019).

### Primers, sequence alignment, RNA analysis, and statistical analysis

All the PCR primers relevant in this study were designed by SnapGene v4.1.9. The graphs were edited by Figdraw,^4^ GraphPad Prism 9.2.0, Adobe Photoshop 2021, Adobe Illustrator 2021, and Excel 2019. A *t*-test (*P* ≤ 0.05) was used to analyze the data from the same treatment under different salt stresses in the wheat and soil samples by GraphPad Prism 9.2.0 software.

## Results and discussion

### The morphological and physiological indexes of wheat growth were improved by KKD1

*Bacillus halotolerans* KKD1 is a member of the *B. subtilis* species complex and exhibits a high degree of conservation and collinearity with the model strain *B. subtilis* 168 (Wu et al., 2021). In our previous study, we confirmed that KKD1 could grow at 13% salinity. It is known that *B. subtilis* possesses plant growth-promoting properties (Xie et al., 2014). In order to determine whether KKD1 could promote the growth of wheat plants, we evaluated wheat plant growth in the presence and absence of KKD1.

**FIGURE 1:**
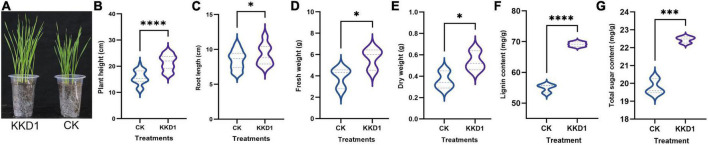
Plant growth promotion effect of strain KKD1 on wheat plants. **(A)** KKD1 promoted the plant growth of wheat; **(B–E)** KKD1 promoted the plant height, root length, fresh weight, and dry weight of wheat; **(F)** KKD1 promoted the lignin content of wheat; **(G)** KKD1 promoted the total sugar content of wheat. Each treatment value is presented as the mean of three replications (*n* = 3) with the standard error (SE). Different letters for each treatment indicate significance between inoculated and uninoculated conditions at the *P* ≤ 0.05 level based on a *t*-test. The * indicates a significant difference (*p* ≤ 0.05); *** indicates an extremely significant difference (*p* ≤ 0.001); **** indicates an extremely significant difference (*p* ≤ 0.0001) compared to the control group.

We observed that *B. halotolerans* KKD1 significantly promoted the seedling and root growth of wheat (Figure 1A). The average shoot length of the KKD1 treatment group was 21.37 ± 2.90 cm, while that of the uninoculated group was 15.74 ± 2.63 cm. The plant height was enhanced by 35.77% in the presence KKD1. Similarly, inoculation with KKD1 caused an increase in wheat root length. The average root length was 9.24 ± 1.46 cm, while the root length without KKD1 inoculation was 8.50 ± 1.30 cm, indicating an increase of 8.70%. In addition, the fresh and dry weight revealed a significant increase in the biomass of wheat when treated with KKD1. The fresh weight and dry weight of wheat inoculated with KKD1 were 5.66 ± 1.00 and 0.54 ± 0.09 mg, respectively, while these values in the wheat without KKD1 were 3.90 ± 0.95 and 0.36 ± 0.08 mg (Figures 1B–E).

The above phenotypic experiments proved that KKD1 is able to positively interact with wheat plants, and to stimulate wheat growth, therefore we speculated that KKD1 could change the wheat physiological indexes positively. As expected, the amount of lignin of wheat inoculated with KKD1 was greatly enhanced by 25.95% compared to the control. Lignin is an important metabolite in plants cell wall that can enhance the mechanical strength of plants, and can affect growth of coleoptiles (Whitmore, 1971). Lignin content of wheat treated with KKD1 was 69.155 ± 0.773 mg/g whilst in the control had only 54.906 ± 1.3637 mg/g (Figure 1F). We found that KKD1 increased the total sugar content of wheat. The KKD1 treatment group contained 22.45 ± 0.178 mg/g, whilst the control group contained 19.78 ± 0.430 mg/g (Figure 1G). In summary, KKD1 improved not only wheat development, but also the physiological indexes of wheat growth.

### *Bacillus halotolerans* KKD1 promoted wheat growth by modulating growth hormone levels

**FIGURE 2:**
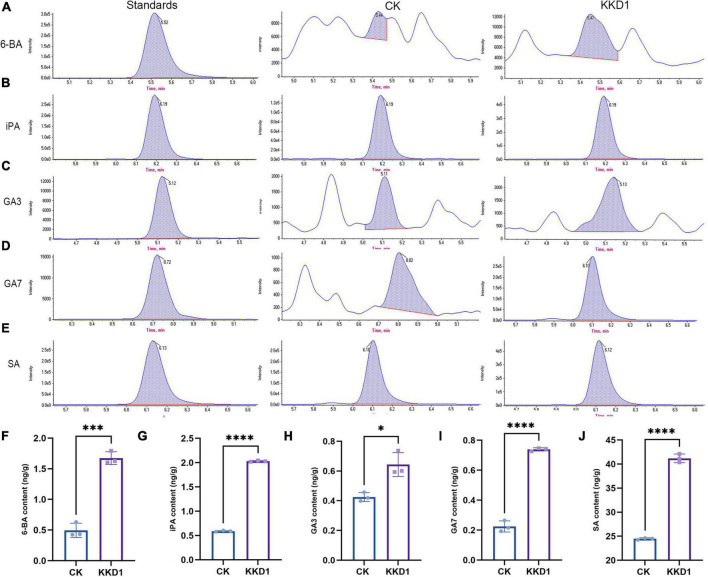
The effect of KKD1 in promoting wheat phytohormones. **(A–E)** KKD1 increased the 6-BA, IPA, GA3, SA, and GA7 contents in the wheat plants; **(F–J)** the contents of plant endogenous hormones in wheat. Each treatment value is presented as the mean of three replications (*n* = 3) with the SE. Different letters at each treatment indicate significance between inoculated and uninoculated conditions at the *P* ≤ *0.05* level based on a *t*-test. The * indicates a significant difference (*p* ≤ 0.05); *** indicates an extremely significant difference (*p* ≤ 0.001); **** indicates an extremely significant difference (*p* ≤ 0.0001) compared to the control group.

To confirm the plant growth-promoting ability of KKD1, we investigated whether the plant endogenous hormone levels were affected by KKD1. 6-BA, a broad-spectrum plant growth regulator, can promote the growth of plant cells and increase the amino acid content (Froldi et al., 1999). The 6-BA content of wheat inoculated with KKD1 was found to be strongly enhanced (1.674 ± 0.102 ng/g) compared to the uninoculated control (0.496 ± 0.117 ng/g) (Figures 2A,F). iPA is a type of adenine cytokinin that can improve plant disease resistance and increase plant sugar content (Keshishian and Rashotte, 2015). The iPA content of the KKD1-treated wheat was also found to be greatly enhanced (2.031 ± 0.013 ng/g) compared to the control containing 0.591 ± 0.013 ng/g (Figures 2B,G). Gibberellin is an essential plant growth promoter that improves plant cell elongation, and GA3 is the most important member of the gibberellin group that can significantly promote the growth of plant stems and leaves (Binenbaum et al., 2018). Our results demonstrated a significant increase in the GA3 content in wheat plants inoculated with KKD1 (0.644 ± 0.081 ng/g), indicating an increase 51.53% compared with the control group (0.425 ± 0.030 ng/g) (Figures 2C,H). Furthermore, the GA7 content of the KKD1-treated wheat was estimated as being 0.739 ± 0.011 ng/g, which was more than three times higher than in the control (0.224 ± 0.037 ng/g) (Figures 2D,I). SA is classified as a plant growth hormone that can improve the resilience of plants (Verma et al., 2016). The SA content of wheat treated by KKD1 was found to be increased by 68.38% (41.20 ± 0.893 ng/g) compared with the control (24.469 ± 0.198 ng/g) (Figures 2E,J).

### The key pathways of *Bacillus halotolerans* KKD1 in promoting wheat growth

**FIGURE 3:**
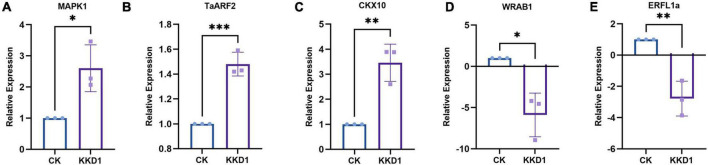
Effects of strain KKD1 application on the expression of growth-related genes in wheat plants. **(A)** MAPK1; **(B)** TaARF2; **(C)** CKX10; **(D)** WRAB1; **(E)** ERFL1a. Each treatment value is presented as the mean of three replications (*n* = 3) with the SE. Different letters at each treatment indicate significance between inoculated and uninoculated conditions at the *P* ≤ *0.05* level based on a *t*-test. The * indicates a significant difference (*p* ≤ 0.05); ** indicates an extremely significant difference (*p* ≤ 0.01);*** indicates an extremely significant difference (*p* ≤ 0.001) compared to the control group.

To elucidate the molecular mechanisms governing wheat growth-related gene expression in the presence of KKD1, wheat genes related to growth metabolism were analyzed by RT-qPCR. The gene expression profiles of these genes were obtained from the wheat seedlings inoculated or not inoculated with KKD1. After 4 h of treatment, the transcriptional expression of target genes in the inoculated group was remarkably different from the control. After KKD1 inoculation, the transcriptional expression of cytokinin CKX10 and auxin TaARF2 was upregulated (1.34–1.48-fold). It is known that mitogen-activated protein kinases (MAPKs) are closely related to diverse biological functions in plants, including plant growth and development under various stress conditions (Dóczi et al., 2012). The MAPK1 in the inoculated wheat seedlings was found to be upregulated (2.6-fold) compared with the control. Ethylene is a gaseous hormone with a wide range of effects in plant growth and development. Increased ethylene content causes the inhibition of plant growth and development (Gamalero and Glick, 2015). Abscisic acid (ABA) is also a plant hormone that inhibits plant growth (Zhu, 2002). The ethylene-related transcription factor ERFL1a and the ABA-inducible protein WRAB1 were significantly downregulated (−2.78 to 5.89-fold) in wheat seedlings treated with KKD1. We conclude that KKD1 improves wheat plant growth mainly by regulating phytohormone levels (Figure 3).

### KKD1 promotes plant growth in various ways

**FIGURE 4:**
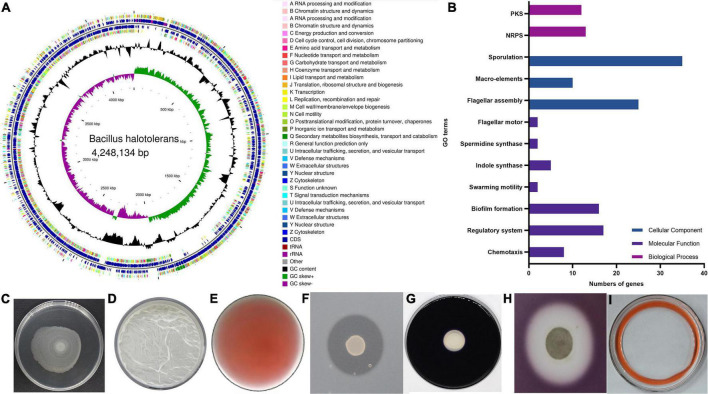
Genetic characteristics and PGP traits of *B. halotolerans* KKD1. **(A)** Genome map of the KKD1 strain in CG view; **(B)** PGP trait-related genes in GO function classification; **(C)** swarming ability; **(D)** biofilm formation; **(E)** IAA production; **(F)** protease production; **(G)** amylase production; **(H)** cellulase production; **(I)** bio-surfactant.

The greenhouse experiments described in 3.1 confirmed that *B. halotolerans* KKD1 possesses PGP properties. To further explore the PGP properties at the molecular level, we analyzed the genomic features of KKD1 (Figure 4A). KKD1 promotes plant growth using three different strategies: colonizing the plant rhizosphere, synthesizing phytohormones, and synthesizing antimicrobial secondary metabolites (Figure 4B).

It is known that colonizing the plant rhizosphere efficiently is crucial for the effectiveness of PGPRs. Rotating flagella are a prerequisite for PGPR to swim in liquid conditions and to swarm on semi-solid surfaces. This motility allows the PGPR to colonize the plant roots and to compete for nutrients and ecological niches (Chen et al., 2007). We detected 29 genes encoding flagellar proteins in the KKD1 genome. Most of these genes were localized in three clusters: 14 genes in the *fli* cluster (*fliDEFGHJKLMPQSTY*), eight genes in the *flg* cluster (*flgBCDEGKLM*), and three genes in the *flh* cluster (*flhABF*). In addition, genes related to the swarming motility (*swrBC*) were also detected in the KKD1 genome (Figure 4C). Biofilm formation is the next step of surface attachment and is essential for root colonization. Extracellular polysaccharides help bacteria to develop the initial biofilm formation morphology (Arnaouteli et al., 2021). The KKD1 genome encodes the *epsA-O* operon (*epsABCDEFGHIJKLNO*), which is involved in extracellular polysaccharide biosynthesis. Moreover, our experimental results also indicated that *B. halotolerans* KKD1 is capable of forming *in vitro* static biofilms (Figure 4D).

Two-component regulatory systems and the Rap—Phr quorum-sensing systems are primary pathways through which *B. subtilis* senses environmental signals and initiates cellular signaling circuits (Zhang et al., 2021). Twenty-nine two-component regulatory systems in *B. subtilis* have been reported to play a key role in physiological processes. As reported, in *B. velezensis* SQR9, the ResD/ResE two-component regulatory system regulates biofilm formation, and the DegU/DegS system was found to be associated with colony architecture, biofilm formation, and root colonization. DegU/DegS and ResD/ResE all exist in KKD1. The Rap—Phr quorum-sensing systems are reported to be involved in sporulation and in the production of secondary metabolites in *B. subtilis* (Bareia et al., 2018). We found eight genes encoding Rap (response regulator aspartate phosphatase) proteins (*rapABCDEFHGI*) and four genes encoding Phr peptides (*phrACGH*) in KKD1.

In addition to colonizing plants roots, PGPR develop their beneficial effects through producing phytohormones. IAA is an auxin that affects plant growth and development. IAA, mainly synthesized from tryptophan in *B. velezensis* FZB42, promotes the growth of *Arabidopsis thaliana* (Idris et al., 2007). The genes *trpA* (tryptophan synthase subunit alpha), *trpB* (tryptophan synthase subunit beta), *trpC* (indole-3-glycerol-phosphate synthase), *trpS* (tryptophan–tRNA ligase), and *abgA* (indole-3-acetyl-L-aspartic acid hydrolase) were detected in the KKD1 genome. We hypothesize that KKD1 uses tryptophan as a substrate for IAA synthesis, and wheat growth is stimulated by bacterial IAA (Figure 4E). Spermidine, a common polyamine, is a pivotal plant-growth-promoting compound. Previous research in our laboratory has shown that spermidine produced by *B. subtilis* OKB105 could promote tobacco root growth (Xie et al., 2014). The same genes *speG* (spermidine acetyltransferase) and *speE* (spermidine synthase) were detected in KKD1.

PGPR produce a variety of enzymes and metabolites (e.g., proteases, amylase, cellulases, and antibiotics) that inhibit plant pathogens and promote plant growth (Ali et al., 2020). A serine protease in *Bacillus licheniformis* W10, identified as an antifungal protein, exhibited potential to control fungal plant pathogens (Ji et al., 2020). Genes (including *paiB, pfpI, nisP, aprE, isp, aprX, pfpI, amyE*) expressing various types of proteases and amylase enzymes were detected in the genome of KKD1 (Figures 4F,G). The cell-wall-degrading cellulase in *Bacillus* spp. can damage pathogenic bacteria and fungi (Radhakrishnan et al., 2017). Glucanase, xylanase, and chitinase (*yaaH, malL, bglA, csn, xynA, xynB, xynC, xynD, gmuG, abfA*) were detected in KKD1 (Figure 4H). Gene clusters encoding Non-Ribosomal Peptide Synthetases (NRPS) and Polyketide Synthases (PKS) also occur in the KKD1 genome. The products of the NRPS gene clusters are the lipopeptides surfactin and fengycin, the siderophore bacillibactin, and the dipeptide bacilysin. The surfactin gene cluster containing *srfA*, *srfB*, and *srfC* was detected in the KKD1 genome. The fengycin gene cluster in KKD1 consists of five NRPs (*ppsABCDE*) (Wu et al., 2021) We extracted the lipopeptide compounds in the KKD1 and detected the bio-surfactant activity by using the expelling oil (Figure 4I). Siderophores are formed under the conditions of low iron availability to enable the uptake of iron ions under such conditions. Bacterial siderophore complexes can provide Fe^2+^ for the plants. The bacillibactin gene cluster contained the *dhbF* and *entABCE* genes. We detected the siderophore ABC transporter permease FecCD and the iron ABC transporter TroD. PKSs catalyze the synthesis of secondary metabolites with antimicrobial action (Fan et al., 2017). The giant PKS gene cluster involved in bacillaene synthesis was present in KKD1. In addition, KKD1 possesses further gene clusters involved in the ribosomal synthesis of antimicrobial peptides (lantibiotics). In summary, KKD1 possesses a large set of genetic features associated with PGP traits (Figure 4 and Supplementary material 4).

### *Bacillus halotolerans* KKD1 improved the wheat growth under salt stress conditions

**FIGURE 5:**
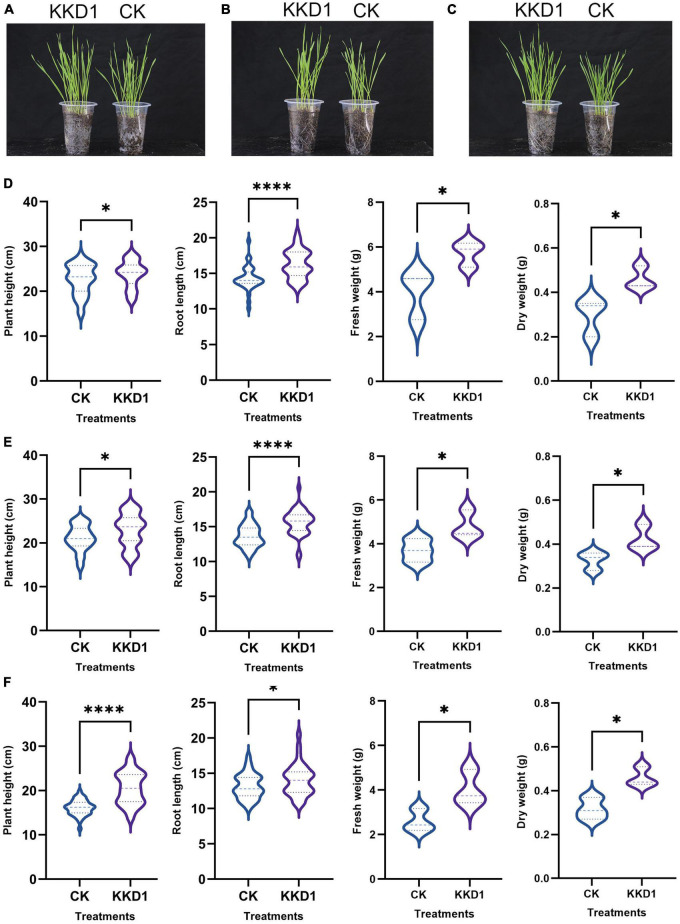
Qualitative effects of *B. halotolerans* KKD1 inoculation on wheat growth under different salt concentrations. [**(A,D)** KKD1 increased the wheat height, root length, fresh weight, and dry weight under 100 mM salt stress; **(B,E)** KKD1 increased the wheat height, root length, fresh weight, and dry weight under 200 mM salt stress; **(C,F)** KKD1 increased the wheat height, root length, fresh weight, and dry weight under 300 mM salt stress]. Each treatment value is presented as the mean of three replications (*n* = 3) with the SE. Different letters at each treatment indicate significance between inoculated and uninoculated conditions at the *P* ≤ *0.05* level based on a *t*-test. The * indicates a significant difference (*p* ≤ 0.05); **** indicates an extremely significant difference (*p* ≤ 0.0001) compared to the control group.

Salt tolerance is usually quantified as plant growth and biomass in saline vs. non-saline conditions. According to our greenhouse experiments, KKD1 promoted wheat growth under different saline conditions. Under 100 mM salt stress, the average shoot height of the KKD1-treated wheat was 23.81 cm, indicating an increase of 5.35% compared with the control group (22.60 cm). Furthermore, in the KKD1-treated wheat, we detected an increase in root length, fresh weight, and dry weight of 14.16, 44.08, and 53.33%, respectively (Figures 5A,D). Under 200 mM salinity, KKD1 also increased the root length (14.32%), shoot length (8.68%), fresh weight (29.92%), and dry weight (27.27%) compared with the untreated control (Figures 5B,E). Similarly, under 300 mM salt stress, the KKD1 treatment enhanced shoot length, root length, fresh weight, and dry weight by 24.52, 6.77, 55.00, and 43.75%, respectively. In summary our data indicated that increasing the salt concentration negatively affected the growth of wheat, while KKD1 could alleviate this phenomenon, at least in part (Figures 5C,F).

### *Bacillus halotolerans* KKD1 alleviates effects of salt stress on wheat growth

**FIGURE 6:**
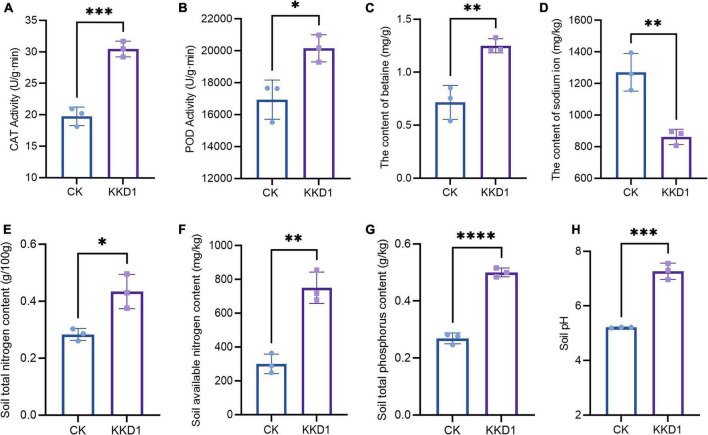
Effect of *B. halotolerans* KKD1 inoculation on wheat growth and soil physicochemical properties under 300 mM salt stresses. **(A)** CAT activity in the wheat seedlings; **(B)** POD activity in the wheat seedlings; **(C)** betaine content in the wheat seedlings; **(D)** Na^+^ concentration in the wheat seedlings; **(E)** total nitrogen content in the soil; **(F)** available nitrogen content in the soil; **(G)** total phosphorus content in the soil; **(H)** soil pH. Each treatment value is presented as means of three replications (*n* = 3) with the SE. Different letters at each treatment indicate significance between inoculated and uninoculated conditions at *P* ≤ *0.05* level based on a *t*-test. The * indicates a significant difference (*p* ≤ 0.05); ** indicates an extremely significant difference (*p* ≤ 0.01); *** indicates an extremely significant difference (p ≤ 0.001); **** indicates an extremely significant difference (p ≤ 0.0001) compared to the control group.

Salt stress leads to osmotic imbalance, ion toxicity, and metabolism disequilibrium, which adversely affect plant growth. PGPR can upregulate key antioxidant enzymes in plants such as POD and CAT, which remove excessive ROS and protect plants from salt stress damage (Liu et al., 2020). Here, we evaluated the ability of KKD1 to mitigate the effect of salinity stress on wheat under 300 mM salt. CAT is a protective enzyme that exists widely in plants. It can remove H_2_O_2_ in plants and avoid oxidative damage to plant cells caused by H_2_O_2_ accumulation (Zhang H. et al., 2022). Under salt stress, the CAT content of the wheat seedlings treated with KKD1 showed a 54% increase compared with the untreated wheat (Figure 6A). POD is an oxidoreductase that can regulate the oxygen concentration in plant cells and eliminate the toxicity of excessive hydrogen peroxide (H_2_O_2_) to plant cells. We found that the activity of POD in the KKD1-treated plants was 20,152 and 16,935 U/(g⋅min) in the control group (Figure 6B). Betaine can maintain the cell osmotic pressure balance and protect plant growth. Under 300 mM salt stress, the betaine content in wheat treated with KKD1 increased significantly and was estimated as 1.25 mg/g, while the control group contained only 0.716 mg/g (Figure 6C). The excessive accumulation of Na^+^ in plant cells could result in the imbalance of osmotic pressure, thus negatively affecting plant health. We found that KKD1 beneficially influenced the export of Na^+^ in wheat cells and maintained the equilibrium of the Na^+^ ions in wheat by decreasing the internal Na^+^ concentration (Figure 6D). Based on the above results we conclude that KKD1 greatly mitigated the adverse effects of salinity stress on wheat and promoted wheat growth under these conditions.

### KKD1 had a beneficial effect on soil properties under saline conditions

Soil physicochemical properties, pH, and the nutritional status play crucial roles in plant growth (Kumar et al., 2021). Representatives of the biocontrol *Bacilli* can remediate soil vitality, improve soil fertility, and accelerate the mineralization of soil nutrients (Li et al., 2021). Here, we compared the physicochemical properties of wheat plants treated with KKD1 or not in the presence of 300 mM salt. We found that at 300 mM NaCl, KKD1 greatly altered the physicochemical properties of the soil.

The total and available nitrogen content are essential indexes of soil health, and so we also determined the nitrogen content of the soil irrigated with 300 mM salt solution (Manzoor et al., 2021). The result indicated that the total nitrogen content of the soil was reduced to 0.263/100 g, while the same soil treated by KKD1 contained 0.434/100 g. The content of the soil-available nitrogen was only 248.715–363.191 mg/kg, while the content of the KKD1-treated soil was 718.114–678.208 mg/kg (Figures 6E,F).

Phosphorus is an essential nutrient element affecting plant growth and development. Traditionally, phosphate fertilizer is applied to improve crop yield and quality. Unfortunately, the long-term application of phosphate fertilizer reduces soil fertility and should be avoided in sustainable agriculture (Yahya et al., 2021). Under 300 mM salt stress, the total phosphorus content of the soil showed the same trend as the total nitrogen content. Compared with the soil without KKD1, the total phosphorus content of the soil treated by KKD1 increased significantly by 85.19% (Figure 6G). After irrigating 300 mM salt solution into the soil, the pH of the soil without KKD1 was lowered to 5.19–5.22, while the soil treated with KKD1 remained at pH 6.96–7.55 (Figure 6H). Therefore, KKD1 could stabilize the acid-base balance of the soil and help the wheat grow in a healthy soil environment. We assume that strain KKD1 may convert insoluble form of P into soluble forms such as H_2_PO_4_ by releasing phosphatases that might be helpful for enhancing soil pH under salt stress (Ali et al., 2021). Besides, the bacterial suspension of KKD1 was alkaline after fermentation, we speculate that may another reason for the change in soil pH.

### *Bacillus halotolerans* KKD1 induces metabolic changes in wheat under salt stress

**FIGURE 7:**
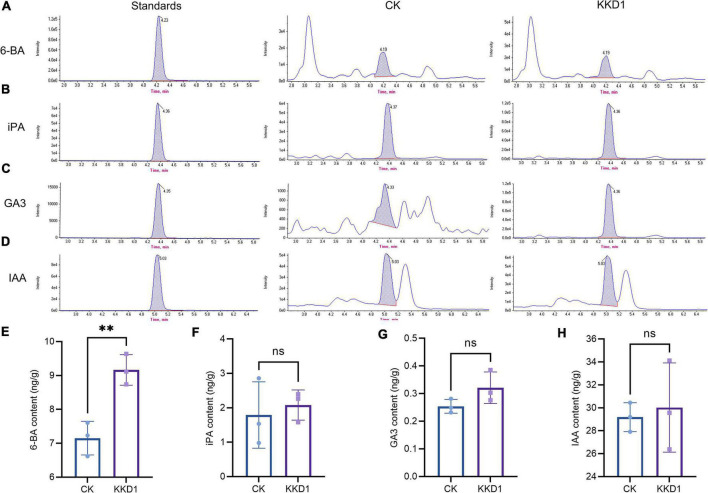
The effect of KKD1 on wheat phytohormone content at 300 mM salt stress. **(A,E)** 6-BA content; **(B,F)** iPA content; **(C,G)** GA3 content; **(D,H)** IAA content. Each treatment value is presented as means of three replications (*n* = 3) with the SE. Different letters at each treatment indicate significance between inoculated and uninoculated conditions at *P* ≤ *0.05* level based on a *t*-test. ns, indicates no significant difference compared to the control group.

Phytohormones play an important role in supporting plant growth and development. Cytokinin, gibberellin, and auxin are three main plant hormones (Yu et al., 2020). We detected and quantitatively analyzed the content of these phytohormones in wheat at 300 mM salt concentration by HPLC-MS. 6-BA, a representative of cytokinin, is known as a plant growth modulator that can influence the division and differentiation of plant cells, promote plant growth, and improve the survival of plants under adverse conditions (Zhu, 2002). The content of 6-BA in the KKD1-treated wheat was 9.16 ng/g as compared to 7.15 ng/g in the control group. GA3, iPA, and IAA are also critical phytohormones affecting growth and the resistance to various biotic and abiotic stress conditions (Zhang H. et al., 2022). Among them, GA3 is a gibberellin, while iPA and IAA are auxins. The HPLC-MS results revealed that KKD1 slightly increased the GA3, iPA, and IAA content of wheat under 300 mM salt stress (Figure 7).

We analyzed the genes related to different salt stress-response pathways by RT-qPCR. Our results revealed that at 300 mM salt stress, KKD1 caused the differential expression of a set of genes involved in wheat salt tolerance. The gene expression profiles of these genes were obtained from three variants, including seedlings inoculated with sterile water and grown in sterilized soil irrigated with 0 mM NaCl sterilized water; seedlings inoculated with sterile water and grown in sterilized soil irrigated with 300 mM NaCl sterilized water; and seedlings inoculated with KKD1 and grown in sterilized soil irrigated with 300 mM NaCl sterilized water.

**FIGURE 8:**
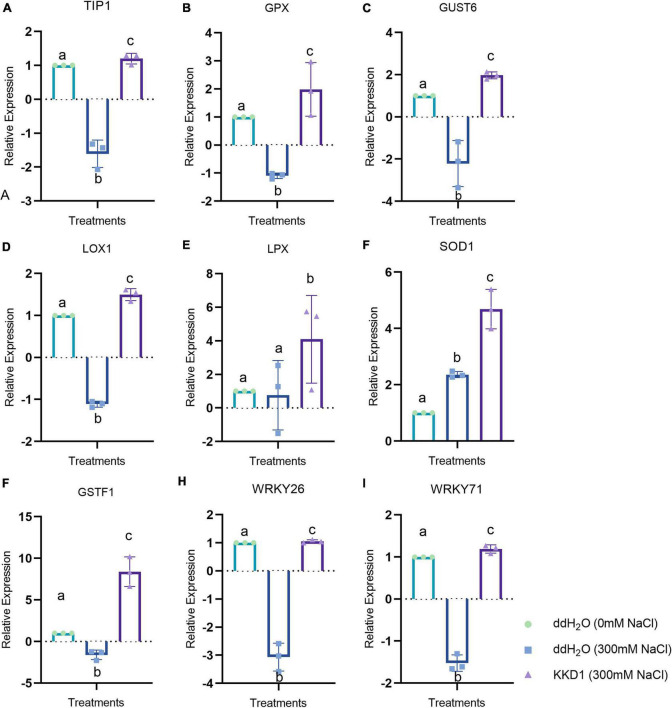
Effects of strain KKD1 application on the expression of growth-related genes in wheat plants under salt stress. (**A**, TIP1; **B**, GPX; **C**, GUST6; **D**, LOX1; **E**, LPX; **F**, SOD1; **G**, GSTF1; **H**, WRKY26; **I**, WRKY71). Each treatment value is presented as means of three replications (*n* = 3) with SE. Different letters at each treatment indicate significance between inoculated and uninoculated conditions at the *P* ≤ *0.05* level based on a *t*-test.

Abiotic stress leads to an imbalance in ROS, which could impair plant cells and affect plant health. Superoxide dismutase (SOD), an enzyme that can enhance the antioxidant capacity of plant cells, eliminates excessive ROS in plant cells and improves the resistance of plants (Jiang et al., 2019). The expression of SOD1 in the KKD1-treated wheat was found to be upregulated by 4.02 times compared with the control. Lipoxygenase (LOX) is an oxidative enzyme containing iron or manganese that catalyzes superfluous hydroperoxide in plants. LOXs are also related to the disease and stress resistance of plants (Mauch et al., 1997). We found that compared with the control group, the lipoxygenase 1 Lpx and Lox1 of wheat treated with KKD1 were upregulated by 1.34∼5.74 times. As antioxidants, plant glutathione transferases are involved in plant development and endogenous metabolism, which is also associated with plant resistance (Taie et al., 2019). Compared with the control group, after inoculating wheat with KKD1 for 4 h, the expression of the glutathione transferase GSTU6 and glutathione POD GPX were upregulated by 4.18∼5.49 times. Proline, as a compatible osmotic solute, can chelate saline ions to maintain the osmotic balance inside and outside of the cell membrane. It is an essential substance required for plants to exist under abiotic stress (Selim et al., 2021). Aquaporin, an integral membrane protein, is capable of discharging Na^+^ from plant cells, thereby avoiding the toxicity of excessive Na^+^ accumulation to plant cells (Wang et al., 2022). Aquaporin TIP1 was upregulated 3.36-fold in the KKD1 treatment group. Ethylene, a gaseous phytohormone, is produced by plants to resist various environmental stresses (Verma et al., 2016). A high concentration of ethylene in plant inhibits plant growth and development. The ethylene-responsive transcription factor ERFL1a of the wheat-treated KKD1 was downregulated by 3.10-fold compared to the control group. The WRKY family of transcription factors participates in the signal transduction pathway of plants to enhance resistance against abiotic stress (Niu et al., 2012). With KKD1 treatment, the expression of WRKY71 and WRKY26 was upregulated 2.57–4.69-fold (Figure 8).

## Conclusion

Soil salinization is a major factor responsible for crop loss in recent years, and alleviating salt stress in crop plants is an important goal of sustainable agriculture (Litalien and Zeeb, 2020). Beneficial rhizobacteria, such as *Bacillus* and *Pseudomonas*, are increasingly used due to their ability to protect plants under adverse environmental conditions (Oljira et al., 2020). *Pseudomonas putida* MTCC5279 mitigated drought stress in *Cicer arietinum* by modulating osmolyte accumulation, ROS scavenging ability, and membrane integrity (Tiwari et al., 2016). Halotolerant *Bacillus velezensis* FMH2 alleviates salt stress on tomato plants by altering physiological and antioxidant responses (Masmoudi et al., 2021). Several PGPR have been identified as being able to alleviate salt stress in plants, but the mechanisms for inducing salt stress tolerance are still elusive.

The beneficial halotolerant rhizobacterium *B. halotolerans* KKD1 was shown to enhance wheat growth. Here, we explored the PGP traits of KKD1 at the physiological and genetic levels. We demonstrated that different growth parameters of wheat, such as (1) seedling morphology, (2) total sugar content, and (3) lignin content, were positively affected. Our result showed that the expression of genes involved in the transcription of phytohormone genes (CKX10, TaARF2, and MAPK1) was upregulated in the KKD1-inoculated wheat. The expression levels of the phytohormones 6-BA, iPA, GA3, GA7, and SA in the KKD1-inoculated wheat were also markedly enhanced. Mining of the KKD1 genome corroborated the rich PGP and biocontrol abilities of KKD1. Numerous genes involved in the colonization of plant roots, synthesis of phytohormones, and synthesis of antimicrobial secondary metabolites were identified (Chen et al., 2020).

The main part of the study was focused on the beneficial function of KKD1 in alleviating salinity stress in wheat plants. Exposure of wheat plants to salinity stress results in ionic stress, osmotic stress, and oxidative stress. These stress conditions reduce wheat growth altogether (Deinlein et al., 2014). KKD1 was able to relieve salt stress by regulating lipid peroxidation, betaine accumulation, and Na^+^ exclusion. The 6-BA level of wheat plants inoculated with KKD1 was markedly improved, while the GA3, iPA, and IAA contents were also slightly improved. In summary, 14 genes participating in plant growth and plant defense were remarkably upregulated in the KKD1-inoculated wheat plants. Their expression pattern corroborated that KKD1 was able to protect plants and regulate plant growth under salt stress conditions. Furthermore, we showed that the presence of KKD1 in the rhizosphere alleviated the poor soil quality caused by high salinity. We found that KKD1 altered some soil physical-chemical parameters, including soil pH, soil nitrogen, and phosphorous content, which were found to be significantly increased.

**FIGURE 9:**
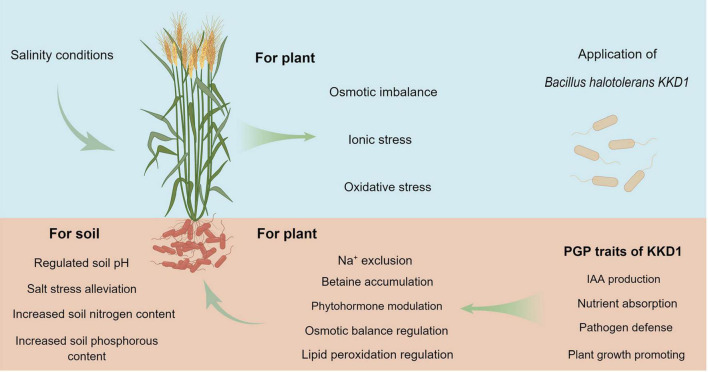
The pathways of *B. halotolerans* KKD1 in alleviating stress in wheat plants under salt conditions.

*Bacillus halotolerans* KKD1 protected plant growth and alleviated salt stress in wheat by modulating phytohormone synthesis and by regulating osmotic balance, ion homeostasis, and gene expression. Based on the beneficial plant-microbe interactions reported herein, it is possible to that KKD1 could be developed as a promising biofertilizer and biopesticide in saline environments to enhance the growth of wheat plants under such abiotic stress conditions (Figure 9).

## Data availability statement

The datasets presented in this study can be found in online repositories. The names of the repository/repositories and accession number(s) can be found in the article/Supplementary material.

## Author contributions

XG and YX designed the study and administrated the project. XW performed the experiments, analyzed the data, and wrote the manuscript. YF performed part of RT-qPCR and pot experiments. RW performed part of qPCR and modified the manuscript. QZ performed part of physiological property experiment. QA modified the manuscript. XW, YF, RW, QZ, QA, HW, QG, RB, YX, and XG revised the manuscript with comments from all authors. RB prepared the final version of the manuscript. All authors read and approved the final manuscript and declare to have no competing interests.

## Footnotes

1
www.majorbio.com

2
http://geneontology.org/

3
https://sg.idtdna.com/scitools/Applications/RealTimePCR/default.aspx

4
https://www.figdraw.com/static/index.html#/
