# Supplementary material for: Bacillus halotolerans KKD1 induces physiological, metabolic and molecular reprogramming in wheat under saline condition
Source: Front Plant Sci. 2022 Aug 11;13:978066. doi: 10.3389/fpls.2022.978066 (PMC9404337; doi:10.3389/fpls.2022.978066)
Supplement: Supplementary file 1 [file Table_1.DOCX]

**Supplementary data 1**

Standard curve of wheat plant metabolite content under normal condition

1. 6-BA

Regression Equation: y = 14549.24955 x + 169.70057 (r = 0.99110)

**
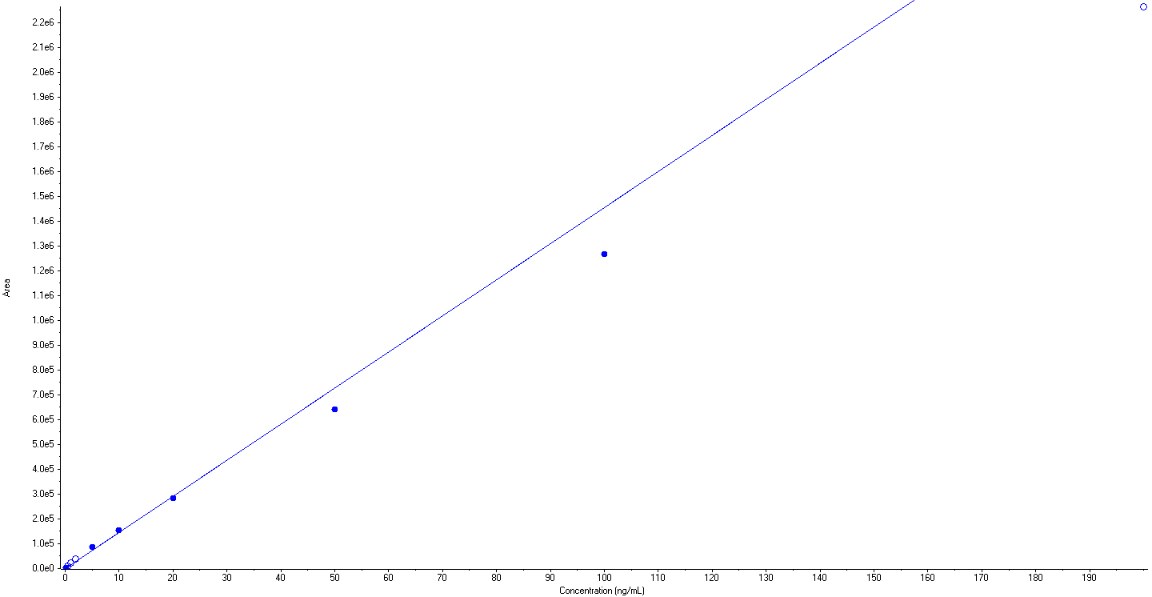
**

1. IPA 1

Regression Equation: y = 3.84220e5 x + 27125.60005 (r = 0.99189)

**
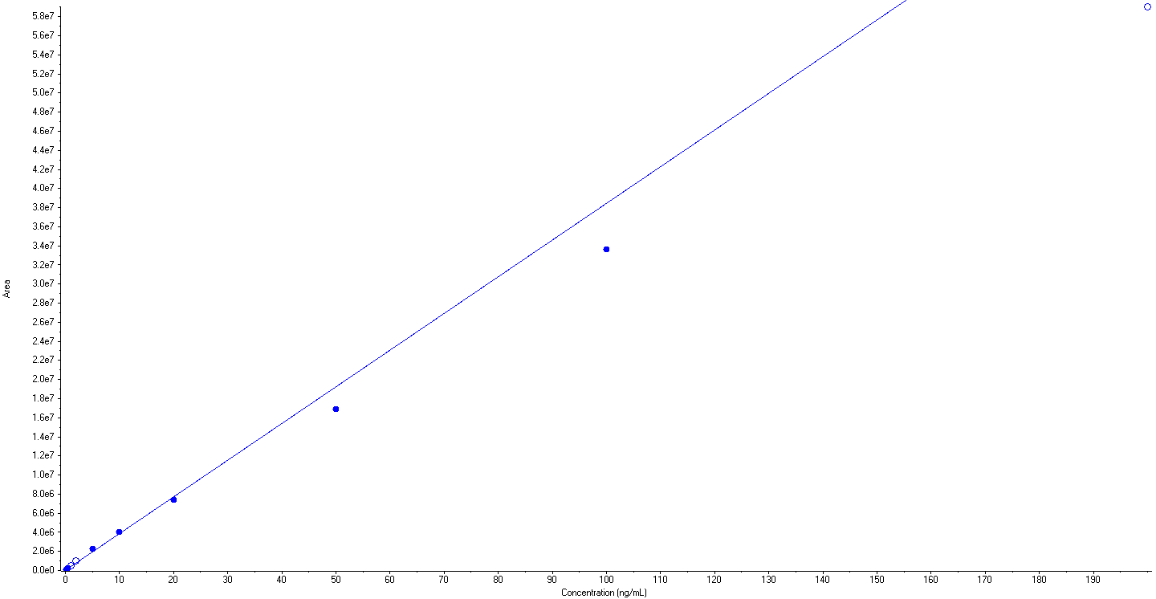
**

1. GA3

Regression Equation: y = 7121.09809 x + 775.88202 (r = 0.99077)

**
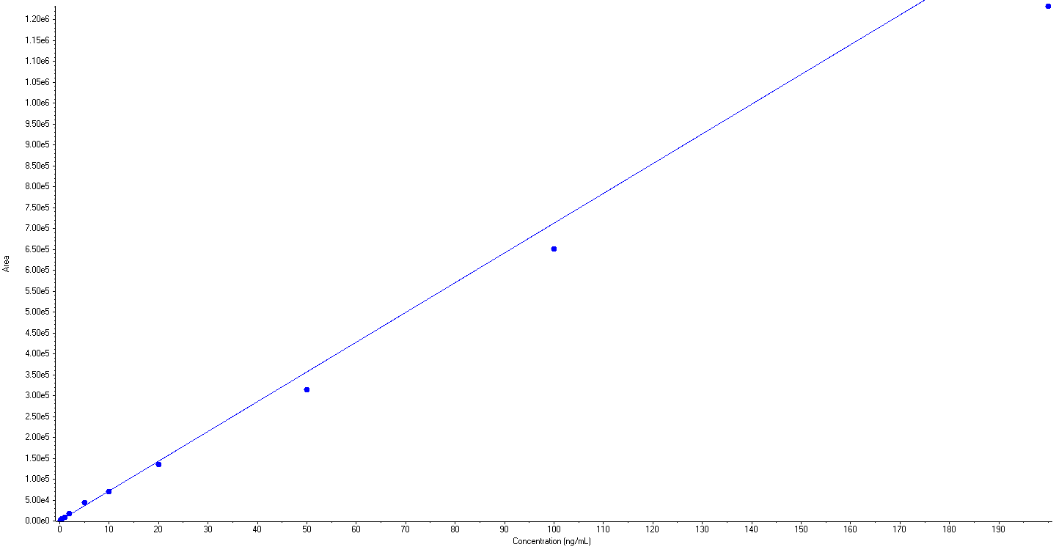
**

1. SA

Regression Equation: y = 22037.83061 x + 4650.80376 (r = 0.99072)

**
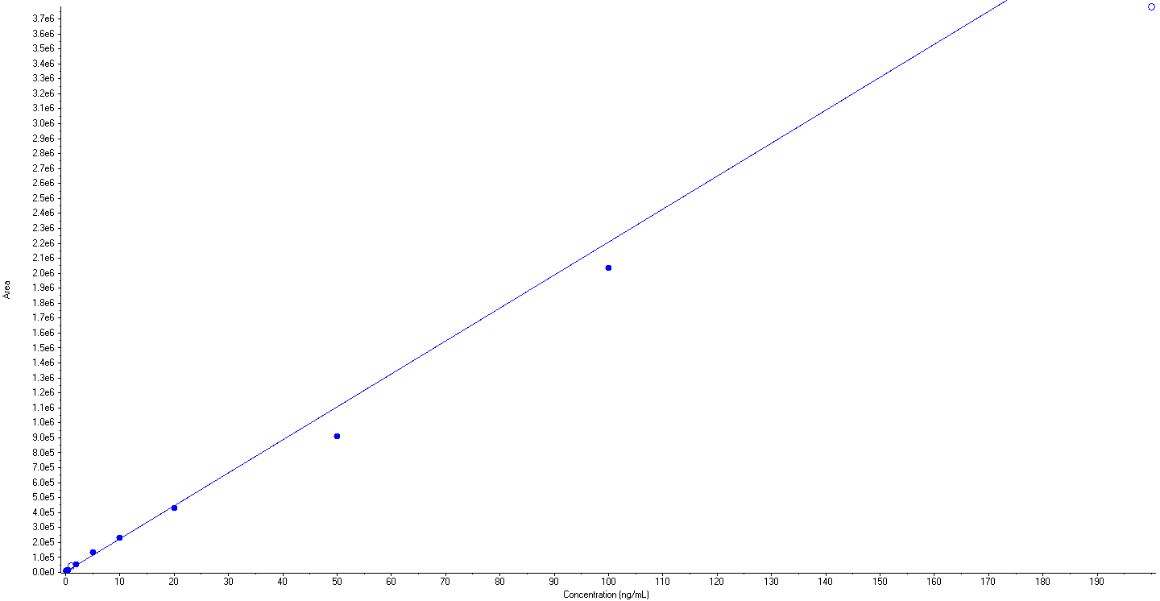
**

1. GA7

Regression Equation: y = 10056.28693 x + 1184.95366 (r = 0.99204)

**
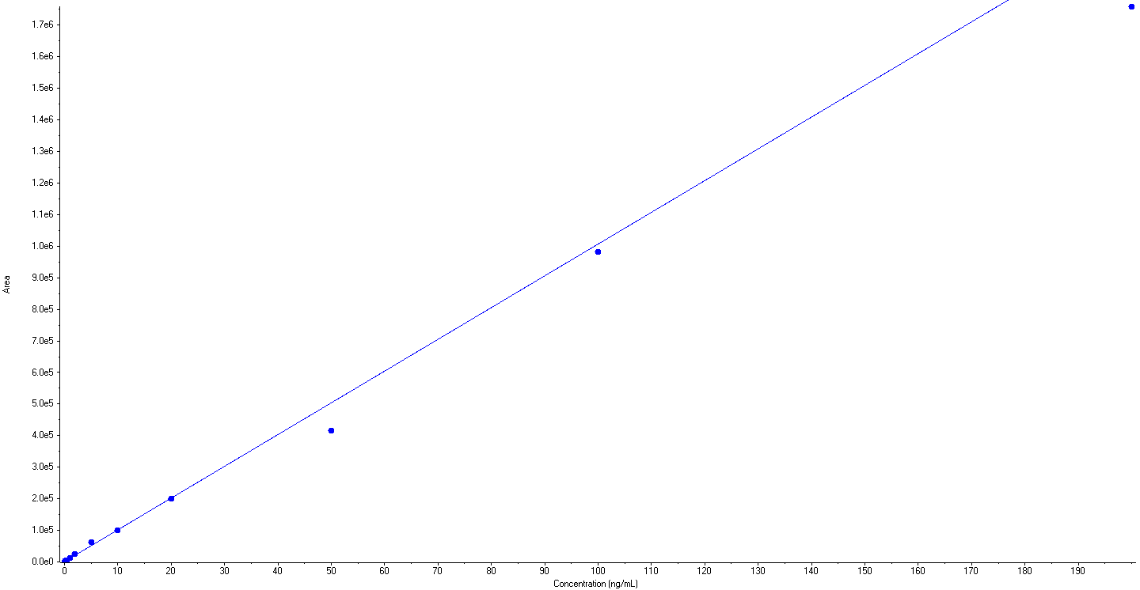
**
