# Supplementary material for: Bacillus halotolerans KKD1 induces physiological, metabolic and molecular reprogramming in wheat under saline condition
Source: Front Plant Sci. 2022 Aug 11;13:978066. doi: 10.3389/fpls.2022.978066 (PMC9404337; doi:10.3389/fpls.2022.978066)

**Supplementary data 2.**

Figure 1. Standard curve of wheat plant metabolite content under salinity condition.

1. 6-BA

Regression Equation: y = 4135.50769 x + 360.51167 (r = 0.99893)

**
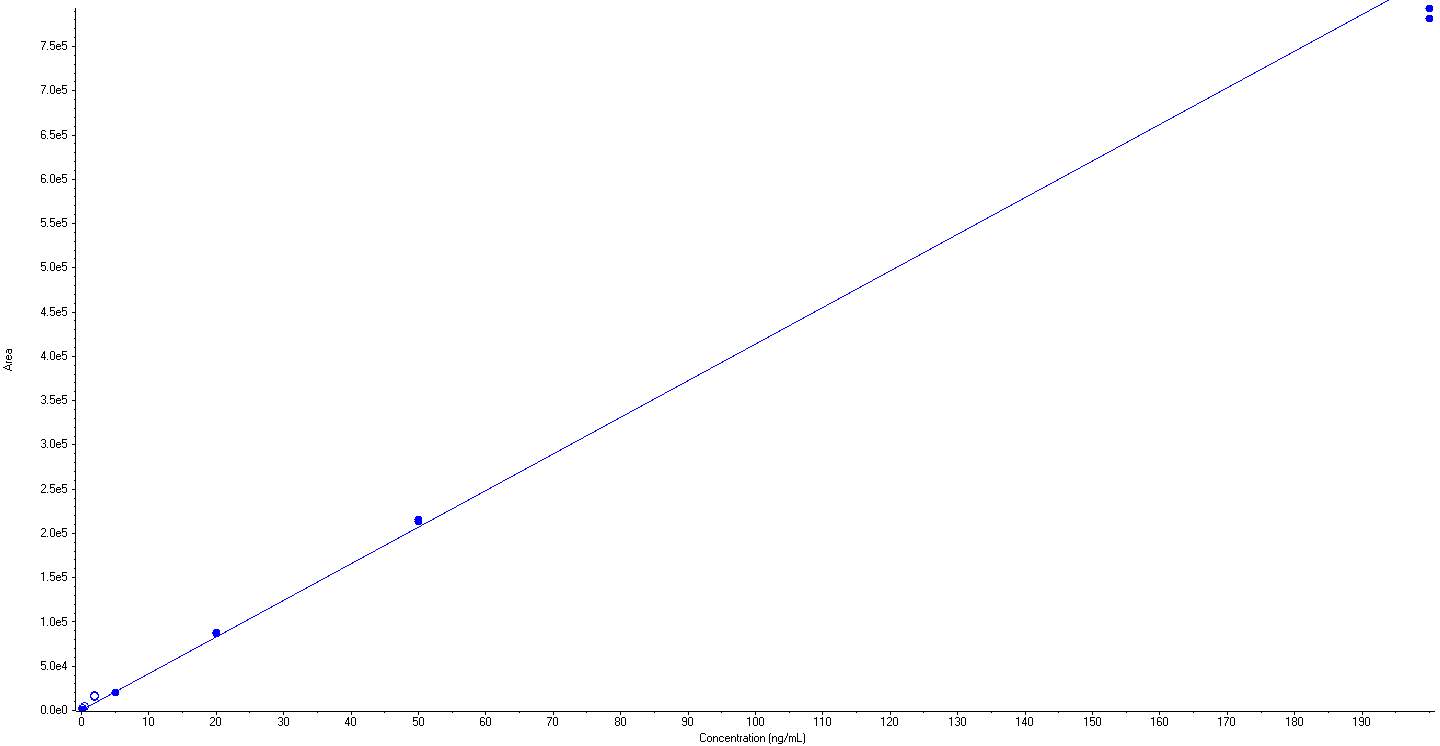
**

1. IAA

Regression Equation: y = 3457.46901 x + -221.74385 (r = 0.99289)

**
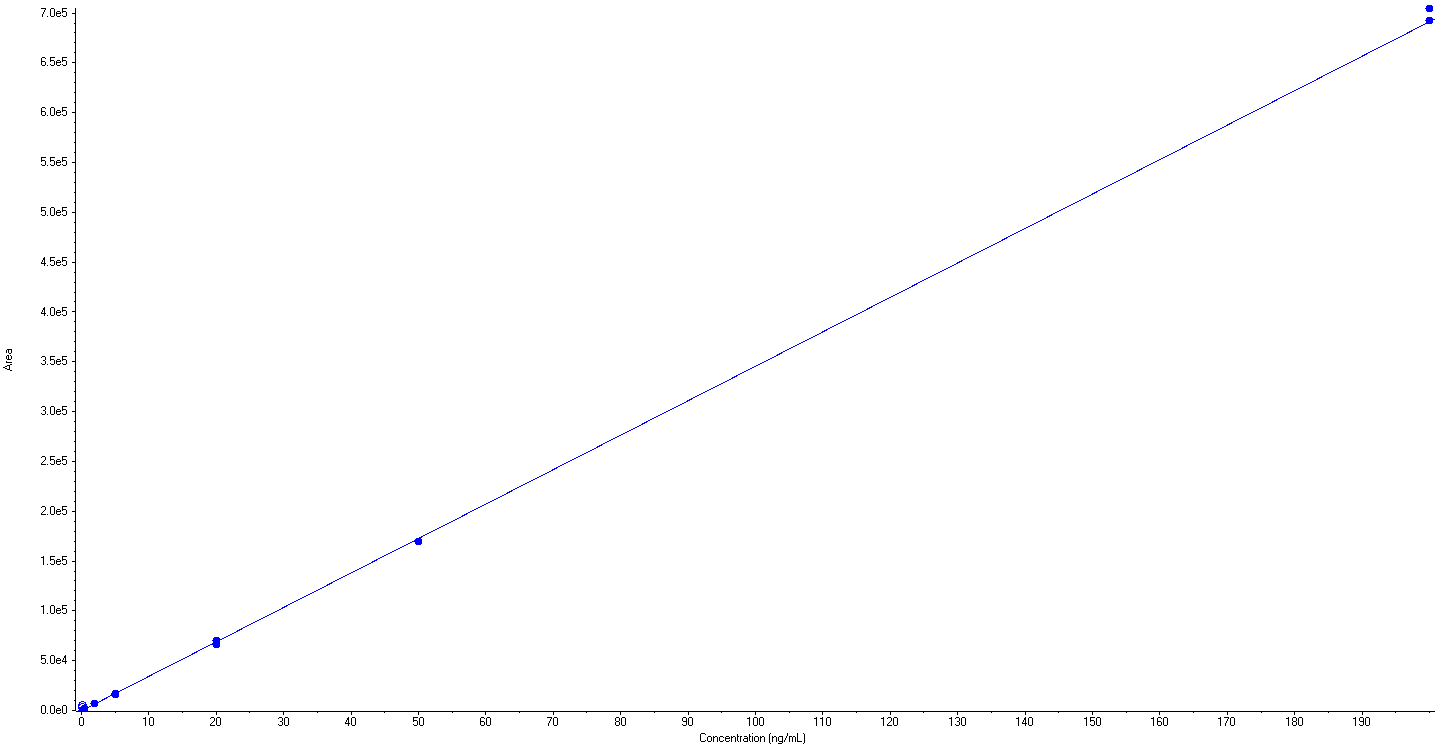
**

1. GA3

Regression Equation: y = 4977.27838 x + -205.88276 (r = 0.99418)

**
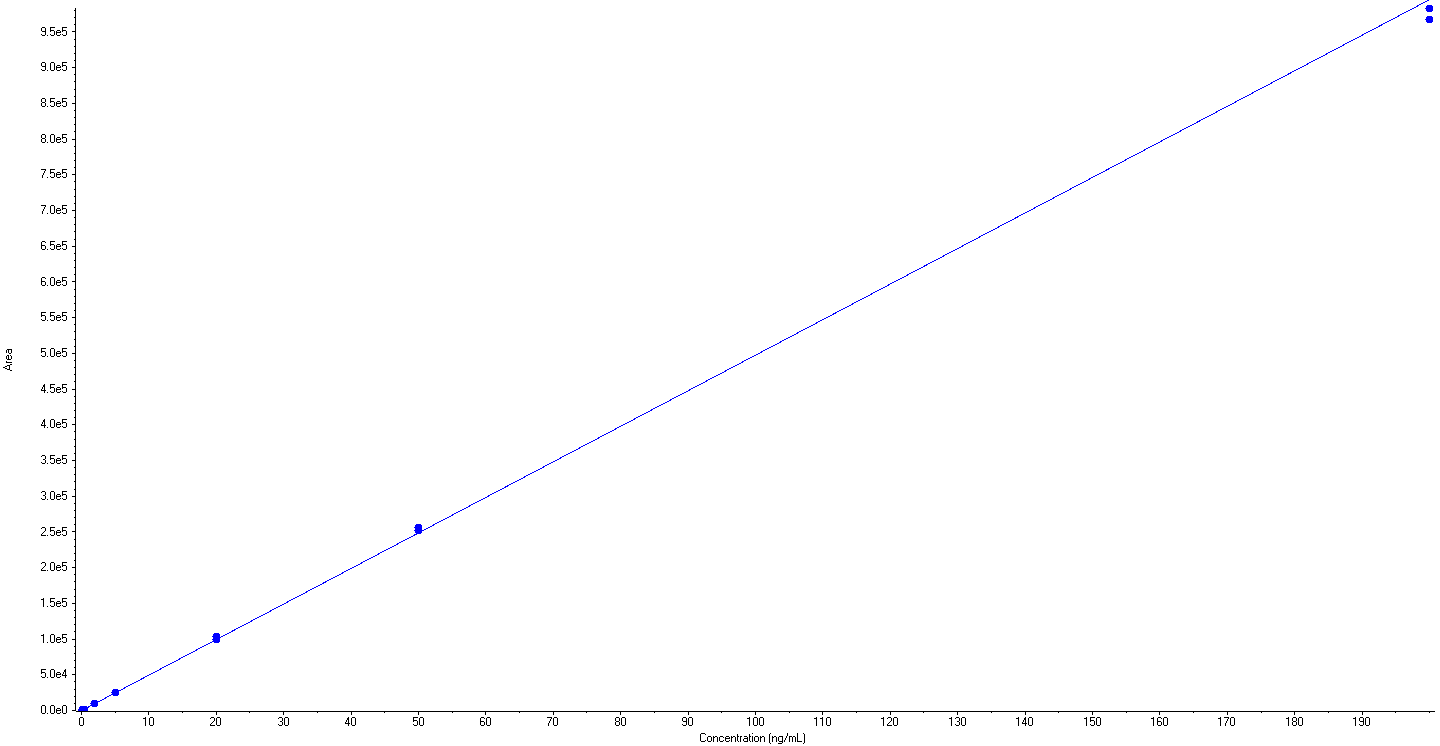
**

1. IPA

Regression Equation: y = 9.15843e4 x + -2526.33011 (r = 0.99623)


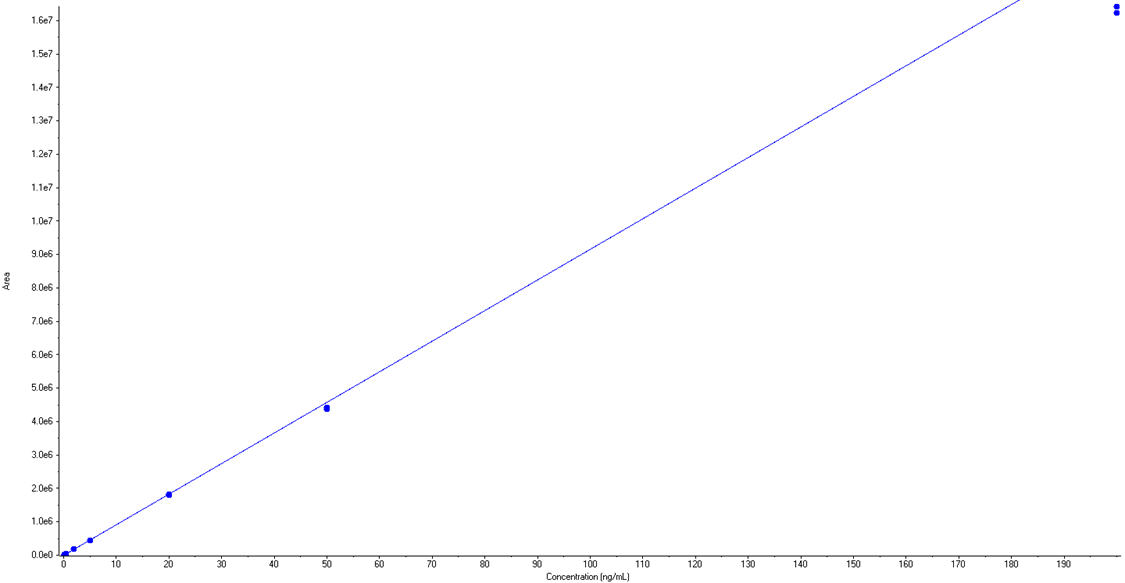

Supplement: Supplementary file 2 [file Table_2.DOCX]
