# Supplementary material for: Bacillus halotolerans KKD1 induces physiological, metabolic and molecular reprogramming in wheat under saline condition
Source: Front Plant Sci. 2022 Aug 11;13:978066. doi: 10.3389/fpls.2022.978066 (PMC9404337; doi:10.3389/fpls.2022.978066)
Supplement: Supplementary file 3 [file Table_3.DOCX]

**Supplementary data 3**

Table 1. The RT-qPCR primers for detection of genes in this study.

| NO. | Oligo Name | Sequence 5’ to 3’ | | Function |
| --- | --- | --- | --- | --- |
| 1 | Actin-F | TGCCCATTTACGAAGGATACG | House keeping | |
|  | Actin-R | GTGTTGGGTTCACAATGTCG |  |  |
| 2 | MAPK-F | CCTACTGGGTCGTTTACTTGC | Mitogen-activated protein kinase | |
|  | MAPK-R | CGAAATTGGATGCCTTGATGG |  |  |
| 3 | TaARF2-F | TTAAGGTGCGTTGGGATGAG | Auxin response factor | |
|  | TaARF2-R | TTGGCACGAGAAAGAGGAAG |  |  |
| 4 | CKX10-F | GCCATTTCAGTTTCCACGAC | Cytokinin dehydrogenase 8 | |
|  | CKX10-R | TCAAGAACACATGCCTCACG |  |  |
| 5 | WRAB-F | GAAGACCGGGCAGATGG | ABA-inducible protein | |
|  | WRAB-R | GTCTTGTCCTTGGCCTCG |  |  |
| 6 | ERFL1a -F | TTCCCATTCAACGGTTACCC | Ethylene-responsive factor-like transcription factor | |
|  | ERFL1a -R | CTGCGGCCTGTTCGTAG |  |  |
| 7 | TIP1-F | CTGCTCTACTGGATCGCG | Aquaporin | |
|  | TIP1-R | CATCACGATCTCCAGCACC |  |  |
| 8 | GPX-F | GACCAACTCCAACTACACCG | Phospholipid hydroperoxide glutathione peroxidase | |
|  | GPX-R | AAACTGATTGCATGGGAAAGC |  |  |
| 9 | GSTU6-F | TTGTTACCAGGGTGAAGCTG | Glutathione S-transferase | |
|  | GSTU6-R | CACTGGGTTGGACTTGAGAAG |  |  |
| 10 | LOX1-F | AGGTGTTCAAGCGGTTCAG | Lipoxygenase 1 | |
|  | LOX2-R | TTGTGATCGGAGGTGTTGG |  |  |
| 11 | LPX-F | GAGGTTTTCAAGCGGTTCAG | Lipoxygenase | |
|  | LPX-R | TTGTGGTCGGAGGTGTTG |  |  |
| 12 | SOD1-F | TCAACTGGACCACACTTCAAC | Superoxide dismutase | |
|  | SOD1-R | CTAGCAACACCATCCACTCC |  |  |
| 13 | GSTF1-F | TGCTGGAGGTCTACGAGG | Glutathione S-transferase | |
|  | GSTF1-R | GCCATGAAGTAGAAGGTGTAGG |  |  |
| 14 | WRKY26-F | TCTTTGGCTTCTCCTTTCACG | WRKY transcription factor 26 | |
|  | WRKY26-R | TGTTGCTCACTTCTACCACTTG |  |  |
| 15 | WRKY71-F | AAACCCGTCATCTCCAAGC | WRKY transcription factor 71 | |
|  | WRKY71-R | TTGTCCTTGGTCACCTTCTG |  |  |
