# Supplementary material for: Bacillus halotolerans KKD1 induces physiological, metabolic and molecular reprogramming in wheat under saline condition
Source: Front Plant Sci. 2022 Aug 11;13:978066. doi: 10.3389/fpls.2022.978066 (PMC9404337; doi:10.3389/fpls.2022.978066)
Supplement: Supplementary file 4 [file Table_4.DOCX]

**Supplementary data 4**

Table 1. Genetic characteristics of *B. halotolerans* KKD1 involved in plant growth promoting

| NO | Function | Gene Name | Gene Description |
| --- | --- | --- | --- |
| 1 | Flagellar assembly | *fliD, fliE, fliF, fliG, fliH, fliJ, fliK, fliL, fliM, fliP, fliQ, fliS, fliT, fliY* | Flagellar biosynthetic and flagellar assembly |
|  |  | *flgB, flgC, flgD, flgE,flgG, flgK, flgL, flgM* |  |
|  |  | *flhA, flhB, flhF, motA, motB* |  |
| 2 | Response regulator | *resD, resE degU, degS* | Response regulator |
| 3 | Phytohormones biosynthetic | *trpA, trpB, trpC, trpS, abgA* | Indole and spermidine synthase |
|  |  | *speG, speE* |  |
| 4 | Antibiotics | *srfA, srfB, srfC, ppsA, ppsB, ppsC, ppsD, ppsE, pksL, pksM, pksN, pksR, pksS, bacA, bacB, bacC, bacD, bacE, bacF, bacG, dhbF, entA, entB, entC, entE* | NRPS and PKS |
| 5 | Protease | *paiB, pfpI, nisP, aprE, isp, aprX, pfpI, amyE* | Protease production |
| 6 | Sporulation | *spo0A, spo0B, spo0F, spo0M, spoIISA, spoIISB, spoIIB, spoIID, spoIIE, spoIIM, spoIIR, spoIIP, spoIIIAA, spoIIIAB, spoIIIAC,* *spoIIIAD, spoIIIAE, spoIIIAF, spoIIIAH, spoIIIAG, spoIIID, spoIVA, spoIVFA, spoIVFB, spoVB, spoVD, spoVK, spoVT, spoVR, spoVS, spoVAA, spoVAB, spoVAC, spoVAD, spoVAE* | Sporulation protein |
| 7 | Biofilm formation | *epsA, epsB, epsC, epsD, epsE, epsF, epsG, epsH, epsI, epsJ, epsK, epsL, epsN, epsO, tapA, sipW* | Extracellular polysaccharides |
| 8 | Regulatory system | *degS, desU, resD, resE, rapA, rapB, rapC, rapD, rapE, rapF, rapH, rapG, rapI, phrA, phrC, phrG, phrH* | Rap–Phr system and Two-component regulatory system |
| 9 | Cellulase | *yaaH, malL, bglA, csn, xynA, xynB, xynC, xynD, gmuG, abfA* | Glucanase, xylanase and chitinase |
